# Supplementary material for: lncRNA-TCONS_00008552 expression in patients with pulmonary arterial hypertension due to congenital heart disease
Source: PLoS One. 2023 Mar 9;18(3):e0281061. doi: 10.1371/journal.pone.0281061 (PMC9997923; doi:10.1371/journal.pone.0281061)
Supplement: Attachment — Submitted filename: Response to Reviewers.doc [file pone.0281061.s008.doc]

List of Responses

Dear Laszlo Farkas and Reviewers:

Thank you for your letter and the reviewers' comments concerning our manuscript entitled " *lncRNA-TCONS_00008552 expression in patients with pulmonary arterial hypertension due to congenital heart disease*" (PONE-D-22-20121). Those comments are pretty valuable and helpful for revising and improving our paper, as well as the important guiding significance to our research. We have studied the comments carefully and have made corrections which we hope meet with approval. Revised portions are marked in red in the Revised Manuscript with Track Changes. The main corrections in the paper and the responses to the editor's comments are as follows:

Responds to the editor's comments:

Comment 1: Please discuss the potential diagnostic and therapeutic implications in more detail in the discussion section of your manuscript.

Response 1: The suggestion was revealing and would provide important information about our manuscript. Partial views have been changed as follows:

1- CXCL14 is the significant differential gene, which has an emerging immune and inflammatory modulator mediated cancer response[35]. CXCL14 association with PAH has not been reported; however, CXCL14's key role in inflammation and immune regulation may be a new therapeutic target for pulmonary hypertension[36]. PAPR-2 is another significantly different gene, and studies have shown that the DNA damage/PARP-1 signalling pathway is important for the development of PAH, but the relevance of PAPR-2 to PAH has not been confirmed[37].

2- Currently, the molecular mechanisms of lncRNA in PAH mainly focus on proliferation, migration and apoptosis of pulmonary artery smooth muscle cells and dysfunction of pulmonary artery endothelial cells[42]. The mechanism of action of lncRNA-TCONS_00008552 was not explored further because the sequence, structure and function of lncRNAs are poorly conserved across species. Despite this, the lncRNA tests are more complex and expensive. However, lncRNAs can be stably expressed in circulation and have the potential to be used as biomarkers, having a significant impact on the treatment and efficacy determination of PAH.

Comment 2:  Please ensure that your manuscript meets PLOS ONE's style requirements.
Response 2: We are very sorry for our incorrect writing. We have modified the manuscript according to the PLOS ONE's style requirements.

Comment 3: Please remove any funding-related text from the manuscript. 
Response 3: We have removed the Acknowledgments Section of our manuscript.

Comment 4: Please state what role the funders took in the study.. 
Response 4: We thank the reviewer for this insightful comment. We have added the Author Contributions. line 386-line 393

Comment 5: Thank you for stating the following in your Competing Interests section. 
Response 5: The authors have declared that no competing interests exist.

Comment 6: PLOS requires an ORCID iD for the corresponding author in Editorial Manager on the paper. 
Response 6: We have updated the corresponding author’s information.

[liaobin@swmu.edu.cn](mailto:liaobin@swmu.edu.cn) (BL) is https://orcid.org/0000-0001-8317-031X.

[594498349@qq.com](mailto:594498349@qq.com) is https://orcid.org/0000-0003-1079-0459

Comment 7: Your ethics statement should only appear in the Methods section of your manuscript. 
Response 7: We have modified the ethics statement(line 98-line 103), and removed any section besides the methods.

Comment 8: Please include your tables as part of your main manuscript and remove the individual files. Please note that supplementary tables.

Response 8: We thank the reviewer for this insightful comment. We have added the tables(line 177-line 179) and supporting information(line 372-line 384) as part of our manuscript.

Comment 9: Please review your reference list to ensure that it is complete and correct.

Response 9: We are very sorry for our incorrect writing that you may have misunderstood. The change has been made in the manuscript.

We tried our best to improve the manuscript and made some changes in the manuscript. These changes will not influence the content and framework of the paper. And here we did not list the changes but marked in red in the revised paper.

We appreciate for Editors/Reviewers' warm work earnestly, and hope that the correction will meet with approval.

Once again, thank you very much for your comments and suggestions.

Sincerely,

Mingbin Deng
